# Perspectives on Developing Healthcare Managers in Africa: The Strathmore Business School's Healthcare Management Programme

**DOI:** 10.3389/fpubh.2019.00044

**Published:** 2019-03-08

**Authors:** Stephen M. Sammut, Ben Ngoye

**Affiliations:** ^1^Health Care Management Department, Wharton School, University of Pennsylvania, Philadelphia, PA, United States; ^2^Institute for Health Care Management, Strathmore Business School, Strathmore University, Nairobi, Kenya

**Keywords:** Healthcare in Africa, healthcare management programmes, healthcare management competencies, healthcare management curriculum, African Institute for Healthcare Management

## Abstract

A substantial shortage of qualified healthcare professionals in Africa continues, but it is now apparent that professionally trained healthcare managers are an equally important need. Health facilities in Africa typically promote physicians into the role of general manager, but physicians and their lay counterparts routinely admit to being ill-prepared for roles as leaders of health systems, healthcare facilities and other services. Few, if any, degree programmes for healthcare management—be they master's in hospital administration or specialized MBA programmes— are available in these regions. And while many master's in public health programmes exist, inclusion of healthcare management content is often an afterthought. This article presents a prototype programme that was designed to address this gap. This comprehensive healthcare management MBA programme that was established at the Business School of Strathmore University in Nairobi, Kenya in 2013 was built around the “Leadership Competencies for Health Care Managers” as promulgated by the International Hospital Federation. The article further presents the development, structure and innovations of the programme, thus providing a blueprint for the development of similar programmes throughout the continent, aimed at addressing the substantial shortage of professionally trained healthcare managers.

## Introduction

To meet the health needs of a rapidly expanding population throughout Africa and the dual disease burden of communicable and non-communicable disease, there is an urgent need for further development of physicians, nurses and other healthcare professionals (1). Likewise, the development of professional healthcare managers is urgently needed at every link in the healthcare value chain, whether they be health facility managers, civic leaders of health ministries, or overseers of public or private payer and insurance systems. Many countries in the developing and developed world plug this gap by promoting health workers—typically doctors or nurses—into management positions. Yet, in the clinical curriculum for physicians and nurses there is little room for exposure to a full range of managerial skill sets. Healthcare professionals seeking to plug this gap and develop or improve their managerial skills often find that the content of general executive education courses miss key elements that support managerial practice and decision-making in healthcare settings. Put differently, the disciplines of leadership and change management, accountancy, economics and finance, human-resource management, operations research and supply-chain management, marketing management, relevant legal and public policy development, and other skill sets are not adequately addressed– and yet they need to be framed in a specialized way for the healthcare environment (2).

Moreover, healthcare management competencies require special consideration when adapting a healthcare management curriculum for different healthcare systems operating in different national and cultural settings. Development of a healthcare management curriculum can be informed by “Leadership Competencies for Health Care Managers” as promulgated by the International Hospital Federation (3). The IHF competencies are derived from the Healthcare Leadership Alliance Competency Directory which in turn reflects work from the American College of Healthcare Executives, the American Association for Physician Leadership, American Organizations of Nurse Executives, Healthcare Financial Management Association, Healthcare Information and Management Systems Society and the Medical Group Management Association. Obviously, these principles have a US-centricity, but they are nevertheless a platform for international application when special circumstances and cultural norms are infused into specific course content.

This perspectives article describes Strathmore University's experience in designing an MBA programme aligned to the globally-relevant competencies, and with immediate relevance to the Kenyan and African healthcare environment.

## Managerial Challenges in the African Healthcare System

Africa bears a significant portion of the global burden of communicable and non-communicable disease. Addressing this dual burden requires strong health systems underpinned by strong leadership, management, and governance. These are however weak in Africa, Kenya included (4–7). Moreover, these weak health systems are further damaged by a critical shortage of health workers occasioned in part by poor retention (7, 8). Yet management capacity is a core component of health systems strengthening (9). And while significant attention has been paid to other elements of the health system such as infrastructure, not enough has been directed toward enhancing the management capacity of those entrusted with improving the functioning of healthcare systems in Africa (10). It is however not clear whether managerial challenges are the same across all countries in Africa.

## Managerial Challenges in the Kenyan Healthcare System

Health services are provided by a mix of private (43%), public (41%), and non-Public non-Private (15%) sector investment (11). However, since 2010, there has been a rapid rise in the role of the private sector in healthcare. This trend was partially stimulated by the formation of several private equity firms focused on healthcare investing. One of these was sponsored by the International Finance Corporation of the World Bank Group and the Bill & Melinda Gates Foundation. The expectations of professional investment managers when staffing the hospitals and clinics in which they invested raised the stakes for the professionalization of healthcare managers.

There were, however, no or few managers with specialized training as healthcare managers. A majority of those managing healthcare institutions learnt on-the-job following their promotion or transfer from clinical practice. And many of these supplemented their learning with either a general master's in business administration, a master's in public health (sometimes with a management concentration), a masters in hospital administration or a combination of these.

Furthermore, Kenya's healthcare system underwent a major transformation starting from 2013 when the country initiated a programme of “devolution” or decentralization as specified in its Constitution of 2010 (7). The Strathmore programme's launch coincided with devolution. The curriculum and course content planning therefore had to anticipate the managerial implications of this change. In particular, devolution had the immediate effect of creating a dramatic need for skilled management for the 47 counties in the country that have essentially autonomous health systems.

## The Strathmore Business School and Healthcare Objectives

Strathmore University is a private institution in Nairobi founded in 1961, shortly before Kenyan independence. Among its Schools is the Strathmore Business School (SBS), founded in 2006. The healthcare management programme (HMP) at SBS was launched in August 2013. The SBS process of developing the curriculum exemplifies the challenges in deriving and customizing a programme to addresses regional needs within a milieu of scarce resources, unique population health needs and a rich cultural context. In the case of SBS, the HMP is structured as a focused MBA, and includes the courses customary to a general MBA, but with those core courses structured around content relating to healthcare. Specifically, the Strathmore HMP addresses: the management and leadership of priority health programmes, health organizations, and multisectoral partnerships; the management systems of health organizations in the public and private sectors, including NGOs and faith-based services; and, the governance and management of health organizations and multisectoral partnerships. The methodology for developing the SBS curriculum is described in the section below.

## Methodology for Creating a Competency-Based Healthcare Management Curriculum in a Developing Country

The IHF competencies are categorized into five critical domains: leadership, communication and relationship management, professional and social responsibility, health and healthcare environment, and business (3). The accepted definitions of each of these in the US setting can be applied but must be modified for any given emerging or frontier market setting. The development of the Strathmore curriculum and course content followed a six-step methodology undertaken from September 2011 through December 2012: 1. Needs analysis through interviews with health ministers, care providers, facility managers, prospective students, payers, and other stakeholders; 2. A search and modification of model curricula outside Africa (there were no healthcare management degree programmes operating in Africa at the time); 3. review and application of the IHF competencies; 4. a high level assessment of cultural issues that might inform content as suggested by Geert Hofstede's cultural dimensions theory as applied to East Africa (12); 5. drafting of a preliminary curriculum with descriptions of course content vetted through rigorous SBS faculty and administrative review together with discussions with external stakeholders, including two focus groups with members of the Health Ministry; and 6. finalization of the curriculum and course content for submission to the Education Ministry of Kenya whose approval for new degree programmes at private universities is mandatory before launch.

It is worth noting that the initial draft of the curriculum was designed around the conventional approach used in most MBA programmes, i.e., 11 or 12 offerings of the traditional disciplines of accounting, economics, finance, organizational behavior, decision analysis, entrepreneurship, marketing and communications, strategy, human resource management, and ethics and governance. Additionally, the healthcare management content was to be provided through six courses specifically on aspects of healthcare offered beyond the core. External vetting and the focus group process, however, exposed strong opposition to this conventional approach by health leaders and prospective students. The input from these stakeholders was that the managerial needs of Kenyan healthcare, and by extension Africa, were so acute that the patients would be best served if the entire curriculum, including the traditional disciplines, was built around healthcare content. Further interviews with the prospective students—working healthcare management professionals—disclosed that course content should be immediately relevant to their current responsibilities. And so, while the designers argued that the advantage of a general MBA curriculum is that it provides a broader theoretical and disciplinary basis to respond to changes in the business environment during the decades of one's career, their argument did not sway opinion.

Furthermore, while the needs analysis indicated that applications from many physicians in managerial roles would be submitted, the programme also had to anticipate that the prospective student body would draw individuals from across the management spectrum including clinical officers, nurses, health administration officers, heads of support units such as radiology, pathology and pharmacy, and information officers. It would also likely include participation from non-clinical managers of health facilities and of NGO operating in the health sector. This diversity of academic backgrounds and professional needs created additional demands that had to be met in the design of the programme.

Moreover, as a former British colony, Kenya's academic mindset from elementary through collegiate education bears major resemblance to the British tradition. There were, therefore, numerous other guidelines from the Education Ministry that informed the structure and design of the curriculum and course content. Among these are that evaluation and grading of students must derive from proctored in-class, closed-book examinations that comprise 80 percent of the final grade. Drafts of the exams are to be reviewed by an Examination Committee within SBS and are graded on a blind basis by both the instructor and an independent invigilator. These practices meant that there would be limited room for term projects and exercises as evidence of competency development. Faculty, therefore, had to design and build testable competencies into the course material and examinations. A second requirement is that graduate level courses are to be based on 45 in-class contact hours. While this requirement provided faculty with a broad canvas to offer comprehensive content, it also limited the number of time-structures available for programme design. An MBA programme in Kenya must also consist of at least 18 courses bringing total class room contact to over 800 h. This surpasses MBA standards in most other countries. Finally, all master's degree programmes require completion of an original research dissertation by each student who must defend the work before a committee. This requirement means that at least one course be dedicated to research skills and it also means that Strathmore needs to allow for sufficient faculty to supervise the dissertations.

The curriculum submitted to the Commission for University Education (CUE) took all these factors into account. And whereas the CUE review process usually takes a year, a provisional approval was issued in < 3 months thus allowing Strathmore to recruit students and launch the programme in August 2013. The rapid approval of the proposed programme, however, created a challenge in recruitment of faculty. Strathmore had expected that 2013 would be available to assemble a slate of domestic and international faculty to teach the full array of courses. The challenges associated with such recruitment at short notice are further described in the section on the African Institute for Healthcare Management.

## Building the Curriculum and Course Content

Based on the above considerations, Strathmore developed a framework that was a hybrid of IHF competencies and conventional MBA content illustrated by healthcare examples. Infusing the course with content that exemplified the Kenyan or East African environment, however, has proven to be a challenge. While the corpus of literature on African health seems infinite, there is a paucity of research on best management practices in the African context, as well as an absence of case studies suitable for teaching. One of the pedagogical objectives of the Strathmore programme has been to develop a body of such literature.

The pedagogical approach that is in place emphasizes the following five domains of competency: The first, leadership, is the “ability to inspire individual and organizational excellence, create a shared vision and successfully manage change to attain an organization's strategic ends and successful performance” (3). Given the subtle concepts surrounding leadership culture, SBS relied on its own Kenyan faculty to develop course material and exercises on leadership rather than risk having international guest faculty build content around Western cultural values.

The second domain of competency is communication and relationship management which are the “ability to communicate clearly and concisely with internal and external customers, establish and maintain relationships, and facilitate constructive interactions with individuals and groups” (3).

The third competency domain is building professional and social responsibility by shaping the “ability to align personal and organizational conduct with ethical and professional standards that include a responsibility to the patient and community, a service orientation, and a commitment to lifelong learning improvement (3).

The fourth competency domain addresses health and the healthcare environment which is the understanding of the healthcare system and the environment in which healthcare managers and providers function (3).

The fifth and final domain of competency is business or the ability to apply business principles, including systems thinking, to the healthcare environment (3).

In addition, the SBS programme imparts the full range of knowledge necessary to guide the ethical operation of healthcare organizations, including accounting, economics, finance, human-resource management, quality management, supply-chain management, organizational behavior, change management and medical sociology. For illustrative purposes, Table 1 maps the various courses and provides an indication of the competency domains that they primarily address.

**Table 1:** Course titles and competency content.

**Knowledge domain**	**Course title**	**Competency domain addressed**
Accounting, Economics & Finance	Financial and Managerial Accounting in Healthcare Organizations	(4) Understanding of healthcare systems (5) Application of business principles
	Financial Management of Healthcare Organizations	(4) Understanding of healthcare systems (5) Application of business principles
	Healthcare Financing and Health Equity	(3) Professional and social responsibility (5) Application of business principles
	Managerial Health Economics	(4) Understanding of healthcare systems (5) Application of business principles
Quantitative and Research methods	Quantitative Analysis and Statistics for Healthcare Management	(4) Understanding of healthcare systems (5) Application of business principles
	Research Methods in Healthcare Management	
	Decision Analysis for Healthcare Managers	
Technology & Innovation management	Healthcare Entrepreneurship and New Venture Management	(1) Leadership (4) Understanding of healthcare systems (5) Application of business principles
	Healthcare Management Information Systems	(2) Communications
	Managing for Quality Patient Care and Efficient Operations	(4) Understanding of healthcare systems (5) Application of business principles
Human & Organizational factors	Organizational Behavior and Change Management in Healthcare Organizations	(1)Leadership (2) Communication/Relationship management (and to limited degree 3, 4, 5)
	Strategic Management in Healthcare Organizations	(1)Leadership (4) Understanding of the healthcare system
	Managing Healthcare Human Resources	(5) Application of business principles
Communications	Marketing Management for Healthcare Enterprises	(2) Communication/Relationship management (4) Understanding of the healthcare system
	Management Communications and Media Relations in the Healthcare Environment	
Health & Society	Healthcare Organization Ethics and Governance	(3) Professional and social responsibility
	National Public and Private Healthcare Systems	(4) Understanding of the healthcare system
	Healthcare Law and Policy	(1)Leadership (3) Professional and social responsibility (4) Understanding of the healthcare system

*Source: Authors' arrangement and International Hospital Federation (3)*.

## Student Backgrounds and Research Requirements

Admission into the Strathmore programme has been competitive. As of the end of 2018, 225 students have been in the programme spread over six cohorts. Of these students 97.3% have been from Kenya, while just under 3% have been from other East African countries. The first four cohorts have graduated. Qualifications for entry are a combination of academic achievement and work experience. Applicants need a minimum of 5 years' managerial experience and must pass the Strathmore's aptitude test, the Graduate Entrance Exam (GEE) or submit recent GMAT results. In addition, candidates must be holders of First-Class or Upper second-class honors degrees in medicine, nursing or other fields related to healthcare or healthcare management from recognized universities. Nearly half of the students who have enrolled are physicians. There has been an equal mix of men and women in the programme. All the students work full-time in managerial roles and spend a total of ten 2-week modules on campus over the 2-year period of course work. The 10 modules are separated by 2-month intervals allowing the students to continue their professional responsibilities as well as to individually immerse themselves in the course content.

A unique feature of the programme is the requirement for an original dissertation. Students begin formulation of a research topic at the start of the programme and design and develop the study throughout the programme's 10 modules. The target date for completion is 6-months following completion of course work, thus meaning that the programme is 30 months in duration.

## Faculty Development and the African Institute for Healthcare Management (AIHM)

Among the obstacles to creating and offering full-scale curricula in healthcare management at institutions of higher learning in Africa is the relative lack of experienced specialty faculty to teach the wide range of subjects that encompass healthcare management. Consequently, faculty development was approached in two ways. First, representatives of SBS, the University of Cape Town (UCT) Business School in South Africa (SA) and the School of Public Health of the University of the Witwatersrand (Wits) in Johannesburg, SA, came together to form the African Institute for Healthcare Management (the “Institute” or “AIHM”) to serve as a consortium of academic institutions and individuals focused on building a pool of world-class faculty, trainers and researchers that can be shared among programmes in healthcare management throughout Africa, whether MBA, MHA, MPA, executive or other programmes. Thus, AIHM seeks to assemble and compensate approximately 20 Fellows at the doctoral level representing the full range of relevant healthcare management disciplines. The Fellows will circulate among African institutions participating in AIHM, and one-third of their time will be protected for research and teaching material development. In this sense therefore, by being members of the Institute, academic institutions could “claim” faculty and benefit from their expertise, without meeting the full cost of having their own tenured or fully employed professors.

Second, and concurrent with the setup of AIHM, SBS has approached the challenge of faculty with a combination of local full-time faculty, practitioner faculty with appropriate academic credentials and international guest faculty drawn from the University of Pennsylvania Wharton School Department of Health Care Management, Bloomberg School of Public Health of Johns Hopkins University, the Harvard Chan School of Public Health, Boston University, the University of North Carolina Gilling's School of Public Health, the Anderson School of Management at the University of California, Los Angeles, the University of Leeds and other institutions. While this approach has proven effective and provides all the opportunities needed for learning by the students, it would be even more effective (and less costly) with a locally-based, specialized and dedicated faculty that focuses on African healthcare issues. Consequently, the interaction between visiting international faculty and local faculty was structured in a way that prepared the local Strathmore faculty to assume full teaching responsibility for most of the courses in the curriculum. This was achieved through observation, co-designing and co-teaching, interactive meetings and learning sessions and granting access to learning materials and faculty development opportunities.

## Other Lessons Learned and Next Steps

Beyond the creativity in sourcing and filling faculty positions for a novel program in a resource-poor setting, one of the key lessons learned has been the highly recursive nature of the development of a competency-based curriculum. The development of this curriculum required several meetings and iterations, with oftentimes a report-back/validation meeting with stakeholders whose input had been sought earlier and whose views might have been modified by the designers in response to other stakeholder concerns. Another lesson is the importance of the students to the development of a continually-improving and perpetually relevant curriculum. As working professionals, the students are acutely aware of the challenges and needs of the population served by the healthcare system. The students and other stakeholders provide SBS with a steady flow of recommendations to enhance the programme. The students have requested more content on leadership, integrating traditional medical care and belief systems into allopathic care, supply chain management and in-depth review of the producer function and its relationship with providers. A Curriculum Committee is devoted to making these changes and submitting them for approval to the Kenyan Education Ministry as well as to the US-based Commission on the Accreditation of Health Management Education (CAHME) for international accreditation.

The SBS programme's next steps are to design and conduct impact assessment of the programme as it relates to health systems improvement, patient care improvements, emerging best practices in the devolved Kenyan health system, career progress of its graduates, development of effective and relevant teaching materials, and incorporation of web-extended learning. At the current time, full time graduate education in Africa carries too large an opportunity cost for the population. Reaching students and providing access to education requires creative approaches in structuring requirements and classroom contact on a part-time basis. Mixed methods and on-line supplements are slowly working their way into the educational process and will accelerate as broad-band and reduced data charges proliferate.

## Conclusion

African healthcare services are at an inflection point driven by many forces such as: 1. the economic advancement of the African population, especially the aspirations of the emerging middle class that seeks better education and healthcare; 2. a reversal of the population diaspora whereby many young Africans who historically had not returned home after medical training abroad are now repatriating and in doing so accelerate improvement in the standards of practice; 3. increased public and private investment in the healthcare infrastructure; and 4. technological opportunities that allow the healthcare system to leapfrog over its developed world counterparts. In response to the growing demand for world-class healthcare, professional management must emerge. Creation of degree programmes supplemented by post-graduate executive education can accelerate the development of managerial depth when built on the experience of Western academic programmes and modified for the special needs and cultures of African peoples.

The case described in this article provides a glimpse as to how higher education institutions could approach the design and development of such a degree programme. Here, SBS built on a US-centric yet globally recognized healthcare management competencies platform, while concurrently embedding the nuances of their African context. The case has also presented two possible approaches to dealing with the real concern of accessing qualified faculty to teach these courses. And it has presented possibilities for programme delivery that can help drive the costs of such programmes down, thus increasing access. While the programme is clearly a work-in-progress, its exposition here should hopefully stimulate action in developing similar programmes to produce suitably qualified and competent professionals that can help lead, guide and manage the health sector challenges facing Africa.

## Author Contributions

All authors listed have made a substantial, direct, and intellectual contribution to the work, and approved it for publication.

### Conflict of Interest Statement

The authors declare that the research was conducted in the absence of any commercial or financial relationships that could be construed as a potential conflict of interest.
